# Elevational Distribution of Flightless Ground Beetles in the Tropical Rainforests of North-Eastern Australia

**DOI:** 10.1371/journal.pone.0155826

**Published:** 2016-05-18

**Authors:** Kyran M. Staunton, Akihiro Nakamura, Chris J. Burwell, Simon K. A. Robson, Stephen E. Williams

**Affiliations:** 1 Centre for Tropical Biodiversity & Climate Change, College of Marine & Environmental Science, James Cook University, Townsville, Queensland, Australia; 2 Key Laboratory of Tropical Forest Ecology, Xishuangbanna Tropical Botanical Garden, Chinese Academy of Sciences, Mengla, Yunnan, P.R. China; 3 Biodiversity Program, Queensland Museum, South Brisbane, Queensland, Australia; 4 Environmental Futures Centre and Griffith School of Environment, Griffith University, Nathan, Queensland, Australia; Consiglio Nazionale delle Ricerche (CNR), ITALY

## Abstract

Understanding how the environment influences patterns of diversity is vital for effective conservation management, especially in a changing global climate. While assemblage structure and species richness patterns are often correlated with current environmental factors, historical influences may also be considerable, especially for taxa with poor dispersal abilities. Mountain-top regions throughout tropical rainforests can act as important refugia for taxa characterised by low dispersal capacities such as flightless ground beetles (Carabidae), an ecologically significant predatory group. We surveyed flightless ground beetles along elevational gradients in five different subregions within the Australian Wet Tropics World Heritage Area to investigate (1) whether the diversity and composition of flightless ground beetles are elevationally stratified, and, if so, (2) what environmental factors (other than elevation *per se*) are associated with these patterns. Generalised linear models and model averaging techniques were used to relate patterns of diversity to environmental factors. Unlike most taxonomic groups, flightless ground beetles increased in species richness and abundance with elevation. Additionally, each subregion consisted of relatively distinct assemblages containing a high level of regional endemic species. Species richness was most strongly and positively associated with historical and current climatic stabilities and negatively associated with severity of recent disturbance (treefalls). Assemblage composition was associated with latitude and historical and current climatic conditions. Although the results need to be interpreted carefully due to inter-correlation between historical and current climatic variables, our study is in agreement with the hypothesis that upland refugia provided stable climatic conditions since the last glacial maximum, and supported a diverse fauna of flightless beetle species. These findings are important for conservation management as upland habitats become increasingly threatened by climate change.

## Introduction

Understanding the links between assemblage structure and environmental factors is fundamental to predicting community responses to climate change impacts. Generally, species’ ranges track climatic envelopes through upward shifts in elevation and poleward shifts in latitude in response to temperature increases from climate change [1].

Elevational transects are often used as surrogates for investigating climate change effects on community patterns [2]. Ecologists sample a given group of organisms across elevational gradients to capture compositional changes in conjunction with environmental variation [3]. In tropical montane habitats, as elevation increases, temperature decreases at a rate of approximately 1°C per 200 meters in altitude [4]. Water availability also increases with elevation due to greater precipitation and a process known as cloud stripping (water deposited from clouds due to the physical interception of clouds and forests), which occurs in the area described as the cloud cap [5, 6]. Changing environmental variables such as these are often associated with high turnover of species across tropical elevational gradients, and subsequently tropical montane habitats are often listed as hotspots of biodiversity [7–9].

As elevation increases, species richness often is reported to display a hump-shaped pattern [10, 11]. This pattern is attributed to either the mid-domain effect (an inevitable outcome when species’ elevational distributions are randomly located within a bounded geographical domain) [12], artefacts resulting from short-term sampling, or greater human disturbance at low altitudes [13, 14]. Alternatively, species richness may decline monotonically with elevation [15, 16], a pattern commonly attributed to changes in environmental factors such as solar energy input, primary productivity and food resources [17–19]. In contrast, some taxa increase in richness with increasing elevation and have been suggested to do so due to lower predation and competition pressures at higher elevations [20–22]. Certain insect groups with soil-dwelling larvae are also suggested to be more species-rich at higher elevations because of a lower likelihood of desiccation in moist soil [23]. Clearly, richness patterns across elevational gradients are taxon-specific and may correlate with a variety of environmental factors.

The Australian World Heritage Wet Topics Area (hereinafter ‘the Wet Tropics’) is characterised by high biodiversity of tropical taxa that are highly structured across temperature/elevation gradients, and is therefore ideal for investigating climate change impacts on a wide range of communities [24]. The Wet Tropics is considered a “mesotherm archipelago” as it is composed of a series of mountain ranges, that generally peak between 1000 and 1600 m a.s.l, forming up to 22 isolated biogeographic and evolutionarily distant subregions [25], separated by lowland valleys or dry areas [24, 26]. While current climatic conditions have been suggested to affect assemblage structure in the Wet Tropics [27], compositional patterns have also been attributed to historical influences from processes such as extinction filtering–where periods of intolerable environmental conditions selectively drove subregional populations to extinction [25, 28], *in situ* evolution within more stable subregions [29, 30], and recolonisation events [29, 31, 32].

The isolated highland areas of the Wet Tropics contain a highly diverse fauna of flightless insect taxa (274 species), 50% of which are restricted to single subregions [29]. As the species richness of these endemic flightless insects is not related to geographical characteristics, such as subregional shape and size that may affect extinction and immigration patterns, these species have been suggested to have evolved *in situ* throughout the Wet Tropics [29]. Flightless ground beetles (Coleoptera: Carabidae) are a major predatory component of the flightless insect taxa in the Wet Tropics. Flightless ground beetles have been suggested to have undergone brachyptery (wing reduction) as an evolutionary response to habitats characterised by high environmental stability [33–35]. Brandmayr [33] specified environmental stability in this context to be due to either dynamic processes, such as the rate of ecological succession across a landscape (where climax forests are strongly favoured by sedentary species), or hydric factors such as the level of soil water balance and risk of inundation (where low risk promotes brachyptery among carabids).

The entire Australian carabid fauna has been estimated to contain a high proportion (~45%) of brachypterous species [36]. Carabids are generally split into groups associated with three main habitat types (vegetation, water bodies and the ground) and in Australia approximately 75% of ground-associated species are thought to be flightless [36]. These beetles constitute an ideal group for monitoring climate change impacts as they not only display high levels of endemism and poor dispersal abilities, characteristic of many other flightless insect taxa in the Wet Tropics, but are also ubiquitous in upland rainforest habitats and relatively easy to sample. Indeed by the 1990’s, new species were rarely being collected by museum taxonomists in this region, indicating that this fauna had been comprehensively sampled [37]. Recent species distribution modelling of this group indicated that the community composition was strongly linked to stable, upland refugial habitats [38]. These habitats are likely to act as microrefugia (small scale refuge areas) [39] where tolerable climatic conditions, and consequently viable populations, were maintained during events such as the Last Glacial Maximum. Staunton *et al*. [38] further suggested that these community patterns supported the time-stability hypothesis, a process whereby habitat stability facilitates the *in situ* evolution of species [40]. Such modelled projections though, have yet to be confirmed by empirical community analyses.

To date, systematically sampled data, from which diversity patterns may be comprehensively analysed and climate change impacts monitored, do not exist for flightless ground beetles within the Wet Tropics. To better characterise the flightless ground beetle community across a spatially heterogeneous portion of the Wet Tropics, we sampled flightless ground beetles (monomorphic brachypterous Carabidae) along elevational transects in five subregions located across a latitudinal extent of approximately 300 km. Initially, we determine the effectiveness of our standard pitfall protocol in sampling flightless ground beetle assemblages. We then examine patterns in species richness and composition across elevational gradients, and link these patterns to current and historical climatic as well as other environmental factors.

## Methods

### Study area

**Fig 1:**
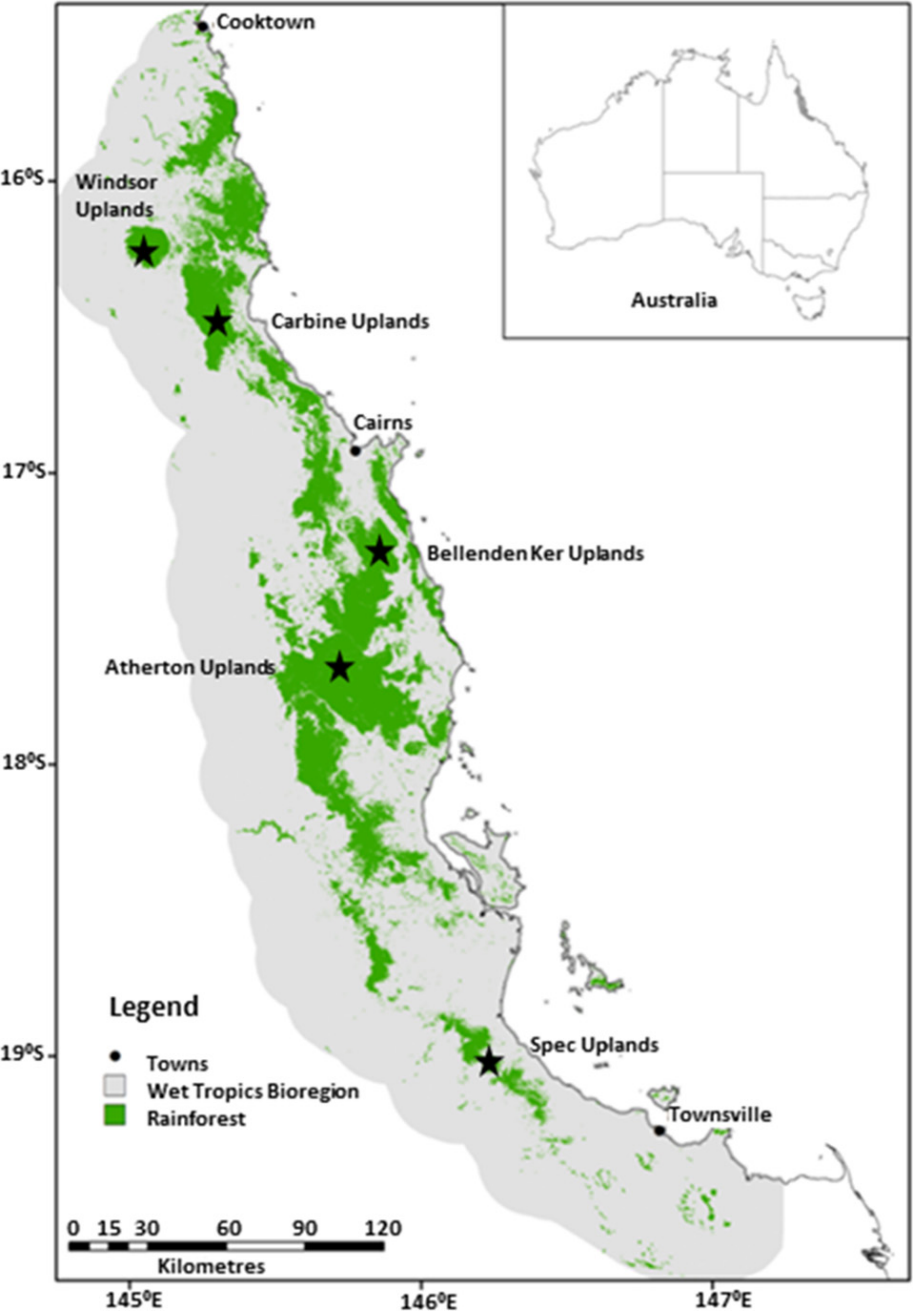
Map of the Wet Tropics. The current extent of rainforest and locations of study sites within five subregions are shown.

This study was conducted in north-eastern Australia (20° to 15°S and 147° to 145°E) within the Wet Tropics, which is approximately 10000 km^2^ in area (Fig 1). The Wet Tropics has been listed as a World Heritage Area since 1988 due to the high biodiversity and endemism of the region’s rainforests. This study was confined to rainforest and covered a range of structural rainforest types across elevational gradients from complex mesophyll vine forest in the fertile lowlands to upland simple notophyll vine forest and, in the case of the Bellenden Ker Uplands, simple microphyll vine-fern thickets above 1500 m a.s.l. [41].

Generally, annual rainfall throughout the Wet Tropics is high (2000–8000 mm per year) [42] with approximately 75–90% of precipitation falling between November and April [43]. Additionally, rainforests located at elevations above 1000 m a.s.l. receive up to 66% of their monthly water input from cloud stripping [5]. Rainfall estimates, therefore, under-estimate total water input. Approximately one-third of the Wet Tropics is higher than 600 m a.s.l., where annual mean temperatures are below 22°C [44].

### Ethics Statement

Specimens in this study were collected from Mt Spec, Atherton Uplands including Wooroonooran National Park, Mt Bellenden Ker, Mt Lewis and Mt Windsor under permit WITK05468508 provided by the Queensland Government Department of Environment and Heritage Protection.

### Experimental design and sampling methods

We collected flightless ground beetles (monomorphic brachypterous Carabidae) from five subregions: Spec Uplands, Atherton Uplands, Bellenden Ker Uplands, Carbine Uplands and Windsor Uplands (Fig 1). Within each subregion, the available elevational gradient within contiguous rainforest was utilised. The extent of the elevational gradient sampled varied between subregions due to differences in the availability and accessibility of rainforest habitats.

Sampling within each subregion was elevationally stratified, with”elevational zones” separated by increments of approximately 200 m of elevation. The elevational zones within each subregion were established by the Centre for Tropical Biodiversity and Climate Change [45] and included: Spec Uplands– 350, 600, 800 & 1000 m a.s.l.; Atherton Uplands– 100, 200, 400, 600, 800 & 1000 m a.s.l.; Bellenden Ker Uplands– 1000, 1200, 1400 & 1600 m a.s.l.; Carbine Uplands– 100, 400, 600, 800, 1000 & 1200 m a.s.l.; Windsor Uplands– 900, 1100 & 1300 m a.s.l. At each elevational zone, sampling was replicated at three plots, separated by ca. 400 m (with the exception of Spec Uplands 350 m a.s.l. where plots were separated by 200 m due to there being only a small patch of rainforest). At Carbine Uplands we were unable to find suitable plots at 200 m a.s.l. due to topographical constraints. At each plot, three pitfall traps, separated by 15 m, were set.

Each pitfall trap consisted of two cylindrical plastic containers (one acting as a sleeve for the other) of diameter 11.5 cm and depth 10 cm. These were set flush with the surface of a soil mound created to prevent flooding of the traps. Traps were protected from rain by a square metal lid (length 26 cm) fixed with wire to a ring of aviary mesh (height 7.8 cm) with 2.5 cm square gaps. Container openings were covered with the same mesh and all mesh was fixed to the trap using wire pegs. Pitfall trap openings were covered with mesh to exclude frogs and skinks. All external components of the traps were metal for protection from rats. Propylene glycol (100%, depth 2.5 cm) was used as a preservative in traps due to its resistance to evaporation and low mammalian toxicity [46]. Some traps in drier locations (e.g. Carbine Uplands 600 m a.s.l. & Windsor Uplands 900 m a.s.l.) had to be reinforced using star pickets and wire to prevent disturbance by feral pigs.

Traps were serviced monthly for between 13 to 18 months, from June 2008 to January 2010. In order to produce directly comparable data sets, based on equal sampling effort, only 12 contiguous months of samples were analysed from each subregion. Additionally, the first month of sampling was not included in any data sets, to account for “digging-in bias” [47]. Data from monthly catches were pooled within a plot. Samples from June 2008 to May 2009 were analysed from Spec Uplands, Atherton Uplands, Carbine Uplands and Windsor Uplands. Bellenden Ker Uplands were unable to be accessed until December 2008, therefore samples collected between January and December 2009 were analysed from this subregion. Of the 2,484 trap catches (3 traps x 3 plots per elevation x 23 elevational zones across the five subregions x 12 months), twenty trap catches were lost in Windsor Uplands 900m a.s.l. and 12 were lost in Carbine Uplands, almost all within the 600 m a.s.l. plots. The loss of trap catches was unlikely to influence the overall results as most trap failures occurred during the dry season when flightless ground beetles were not caught in functioning traps at 600 m a.s.l. in Carbine Uplands or other elevations in Windsor Uplands.

Flightless ground beetles were extracted from samples and sorted and identified to species level in collaboration with Carabidae expert Geoff Monteith of the Queensland Museum, Australia. Nomenclature used derived from works of Sloane [48] and Darlington [36, 49–53]. Where a species had not been formally identified, morphospecies codes were assigned to be consistent with those used in the Queensland Museum. A reference collection of identified species has been deposited in the Queensland Museum.

### Environmental data

We generated a total of 17 predictor variables that are likely to explain the variation in diversity and assemblage composition of flightless ground beetles, based on other local and international studies [33, 54–58]. Using the Anuclim 5.1 software [59], we generated the following bioclimatic variables: annual mean temperature (abbreviated as “Ave.T”); temperature seasonality (“Seas.T”); maximum temperature of warmest period (“Max.T”); minimum temperature of coldest period (“Min.T”); annual precipitation (“Ann.P”); precipitation seasonality (“Seas.P”); precipitation of wettest quarter (“P.Wet.Q”) and precipitation of driest quarter (“P.Dry.Q”). These variables have been strongly linked to vertebrate and dipteran distributions in the Wet Tropics [55–57]. Climate data were spatially derived from ANUCLIM [59] at a 9-second resolution (approximately 250 m grids). ANUCLIM data sets consist of values that are 30-year averages and the most recently produced data sets (averaged over 1976–2005) were chosen for this study.

Two soil moisture layers were incorporated into our analyses due to the strong relationships flightless ground beetles are suggested to have with this climatic factor [33]. First, a soil plant available water holding capacity (“AWC”, this quantifies soil’s ability to retain water) layer was sourced from the Soil and Landscape Grid of Australia, which was based on estimated values from 0-5cm depth and derived from the National Soil Attribute Map as a composite product (http://www.clw.csiro.au/aclep/soilandlandscapegrid). Second, a topographic wetness index (“TWI”, an index that quantifies topographic control of hydrological processes) layer was sourced from the Commonwealth Scientific and Industrial Research Organisation and the Terrestrial Ecosystem Research Network, created using terrain analysis techniques developed by Gallant [60] (http://www.asris.csiro.au/arcgis/rest/services/TERN).

The local habitat heterogeneity (“Habitat”) of forest floor structures was investigated across 10 m × 50 m transects at each plot due to the strong relationships flightless ground beetles are suggested to have with this habitat characteristic [54]. The proportion of forest floor within this transect covered by rocks, logs and buttress roots, was estimated and recorded once at each plot between 2008 and 2009 using a scale from 0 to 4, where 0 = absent, 1 = 1–24%, 2 = 25–49%, 3 = 50–74% and 4 = 75–100%. The same scale was used to estimate recent disturbance due to tree falls (“Treefall”), largely influenced by a recent cyclone (Tropical Cyclone Larry 2006). Additionally, fine-litter standing crop (“Litter”) was measured using a volumetric device, comprised of a compression cylinder and stick [61], from two plots per elevational zone during the 2008–09 wet season (mean of these two values were used for each elevational zone). A layer indicating aspect (the direction in which a land surface slope faces–expressed as degrees from north) was obtained from Geoscience Australia (http://www.ga.gov.au/) which generated the data from the Smoothed Digital Elevation Model (DEM-S; ANZCW0703014016), which was derived from the 1 second resolution SRTM data acquired by NASA in February 2000. Layers indicating elevation and latitude were also sourced from Geoscience Australia.

An historical vegetation layer (“Hist.veg”) was sourced as a product created and published by Graham *et al*. [58]. According to Graham *et al*. [58], this layer displays the spatial variation in historical habitat stability throughout the Wet Tropics by projecting spatial changes in vegetation distribution back 18,000 years before present, assuming that the vegetation dispersed at a rate of 20 m y^-1^ [58]. We chose the layer with a vegetation dispersal rate of 20 m y^-1^ (compared to their other products representing no dispersal and rates of 5 m y^-1^ and 10 m y^-1^), as this product was suggested to best explain species richness of vertebrates and invertebrates throughout the Wet Tropics [58]. To create these layers, these authors applied the Viterbi algorithm [62], which uses dynamic programming, to reveal the likely steps through historical time periods that resulted in rainforest existing at specific locations on a map. These calculations enabled the historical stability of areas to be measured throughout the Wet Tropics, relative to the modelled vegetation dispersal rates [58].

### Data analysis

We first tested our sampling sufficiency within each subregion, using individual- and coverage-based rarefaction curves [63]. Individual-based rarefaction curves plot the number of species against a given number of individuals taken randomly from the observed data. Extrapolation of individual-based rarefaction curves allow us to estimate the number of species which would have been captured if we had collected more beetles. Coverage-based rarefaction curves, on the other hand, plot the sample coverage, which is a measure of sample completeness (defined as “the proportion of the total number of individuals in a community that belong to the species represented in the sample” [63]), against a given number of individuals. Extrapolation of coverage-based rarefaction curves predicts if and how much increased sampling intensity can improve sample completeness. Rarefaction curves were drawn using *iNEXT* package ver. 1.0 [64] available in *R* statistical software (ver 3.1.0.) and extrapolated by a factor of two.

Furthermore, we compared our observed species richness with the number of species recorded from previous surveys of the same subregions (data available from Queensland Museum database). Additional locality records of species of the genus *Feronista* were supplied by Kipling Will (University of California, Berkeley). As opposed to rarefaction curves which assess sample completeness of the beetles which could be captured using a given sampling method (pitfall trapping), these historical records allowed us to assess how much our fully standardised, quantitative dataset represent the entire flightless beetle fauna within each subregion, recorded to date.

We tested whether flightless ground beetle species richness and abundance changed with elevation. As coverage-based rarefaction curves indicated our sampling was comprehensive (>99%, see Results) species richness estimators were not used. We fitted generalised linear mixed models with Laplace approximation to count data, using the *lme4* package in *R* software. Subregion was included as a random factor and negative binomial distributions were utilised to account for overdispersion.

We also tested whether assemblage composition was elevationally stratified using permutational multivariate ANOVA (PERMANOVA), available from *PRIMER6* and *PERMANOVA+* software [59]. Actual elevations at each plot were incorporated in the analysis as covariates and subregion as a random factor. Type I sums of squares (sequential fit) were used to calculate pseudo-*F* values, and *P* values were calculated using 4999 permutations of residuals under a reduced model [65]. We first fitted subregion (random factor) and then elevation (covariate) in the model. Beetle abundances were square-root transformed for all multivariate analyses.

We visually investigated variation in ground beetle assemblage composition among plots, using a non-metric multi-dimensional scaling (NMDS) ordination. Multidimensional scaling calculates distances matching dissimilarities between points, in this case plot assemblages, in multi-dimensional space–the final solution of which is projected onto two or three dimensions for ease of interpretation [66]. We generated an NMDS ordination, using the *vegan* package (ver. 2.3–4) in *R*. All multivariate analyses were performed using Bray-Curtis dissimilarity index with a dummy variable added to all plots (value of 0.0000001). A dummy variable was added to generate ecologically meaningful dissimilarity values when the samples were depauperate, i.e. they consisted of very few or no individuals [67].

We further assessed which environmental variables other than elevation *per se* were likely to explain variation in species richness and species composition of ground beetles using an information theoretic approach [68]. We fitted generalised linear models (for species richness) and multivariate GLMs (for assemblage composition), developed by Wang *et al*. [69], using 12 selected predictor variables (see selection process below). We adopted a model averaging technique, which quantified the relative importance (likelihood) of each of the predictor variables based on all of the possible models that can be generated using combinations of 12 predictor variables (2^12^ = 4096 models). We used a modified Akaike Information Criterion (AICc), as the number of samples was relatively small compared with the number of predictors. We first calculated the Akaike weight of each model, which represents its relative importance compared to other models. The relative importance of each predictor variable was quantified by summing the Akaike weights of all models in which that predictor variable was included. Instead of using an arbitrary cut off value to reduce the number of candidate models (using ΔAkaike), we included all possible 4,096 models to calculate the sum of the Akaike weights. We selected ‘plausible’ predictor variables by testing whether the sum of the Akaike weights of each predictor variable was significantly greater than the summed Akaike weights obtained from a series of null datasets generated by permuting the samples. We compared observed summed Akaike weights with those derived from 999 null datasets. Finally, we calculated the standardised effect size of each predictor variable by calculating the differences between observed summed Akaike weight and mean summed Akaike weight derived from the null datasets, divided by the standard deviation of the summed Akaike weights of the null datasets. The above calculations were all executed using the *mglmn R* package developed by Katabuchi and Nakamura [70].

Before executing *mglmn*, we reduced the number of predictor variables from 17 to 12 by examining all pair-wise correlations (S1 Table). If two predictor variables of similar properties (e.g. related to temperature or precipitation) were highly correlated at r > 0.90, we removed one of them. We included latitude as a predictor variable, as this may explain spatial relationships of the subregions (which are spread latitudinally). Although the number of predictor variables was large relative to that of samples, the use of the AICc value reduces the risk of over-parameterization as this ‘penalises’ over-parameterised models, giving low Akaike weights, as demonstrated by Nakamura *et al*. [71].

Linear regressions were performed using the *visreg* package (ver. 2.2–2) in *R*, to investigate the nature of relationships between observed species richness and the four predictor variables indicated by the information theoretic approach (see Results), as likely to explain variation in this richness pattern. Additionally, the five predictor variables indicated by the information theoretic approach as most likely to explain variation in the community composition of flightless beetles were overlaid onto the ordination using the *vegan* (2.3–4) and *MASS* (ver. 7.3–45) packages in *R*.

## Results

### Taxonomic summary

A total of 4529 flightless ground beetles were captured. These belonged to 16 genera and 43 species, including 14 (33%) species known to be subregional endemics (S2 Table). Tribes dominating the overall fauna included Pterostichini (28 spp., 2559 individuals), Pamborini (3 spp., 1113 individuals) and Ozaenini (1 sp., 740 individuals). The most abundant species was *Notonomus montorum* representing 18% of the total individuals, followed by *Mystropomus regularis* (16%) and *Pamborus euopacus* (14%). With samples from all five subregions combined, there were relatively few rare species with only five singletons and two doubletons. Individuals from Bellenden Ker Uplands accounted for more than half the total abundance of ground beetles sampled. Observed species richness was about two and a half times greater at Atherton Uplands and Bellenden Ker Uplands than Spec Uplands and Windsor Uplands (Table 1). The greatest overlap of species between any two subregional assemblages was found in the Atherton and Bellenden Ker Uplands. However, these subregions also contained the highest numbers of subregionally endemic species (S2 Table).

**Table 1:** Numbers of shared species between subregions (below the diagonal) and the total numbers of species for each subregion with number of unique species to the subregion in parentheses (in the diagonal). All values are based on observed sample data from: Spec Uplands (SU), Atherton Uplands (AU), Bellenden Ker Uplands (BK), Carbine Uplands (CU) & Windsor Uplands (WU).

	SU	AU	BK	CU	WU
SU	**6 (3)**				
AU	2	**16 (9)**			
BK	1	5	**16 (11)**		
CU	2	2	1	**13 (8)**	
WU	2	1	0	3	**6 (2)**

### Sampling effort

**Fig 2:**
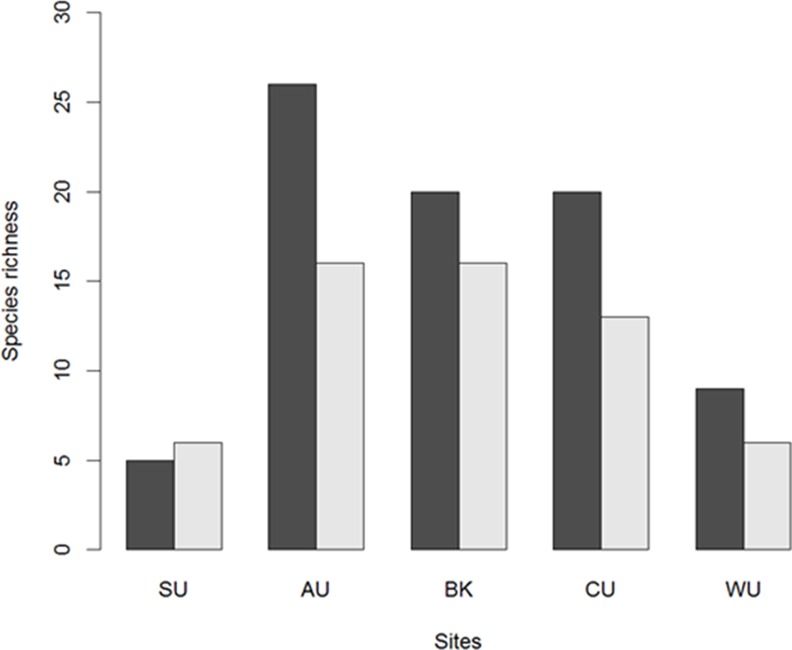
Species richness of flightless ground beetles in the Wet Tropics recorded previously (charcoal bars) and this study (light grey bars). SU = Spec Uplands; AU = Atherton Uplands; BK = Bellenden Ker Uplands; CU = Carbine Uplands & WU = Windsor Uplands. A new species was recorded at Spec Uplands (*Castelnaudia* sp.1).

Of the 61 flightless ground beetle species previously recorded within these five subregions of the Wet Tropics, 43 (70%) were collected in this study. The majority of species known to occur within each subregion were also collected by our methodology (Fig 2). Species that were not sampled in our study were predominantly from the genera Feronista, Leiradira and Notonomus. Despite the absence of some species, rarefaction and extrapolation curves clearly suggested that increased sampling effort using our pitfall methodology would not have yielded a substantial number of additional species. Individual-based rarefaction curves were stabilising or had reached an asymptote at the end of observed number of individuals for all but one subregion (Carbine Uplands where extrapolated number of species continued to increase; S1 Fig). Furthermore, coverage-based rarefaction curves indicated that a sample coverage of greater than 99% of species was attained for all subregions (S2 Fig).

### Elevational patterns

**Fig 3:**
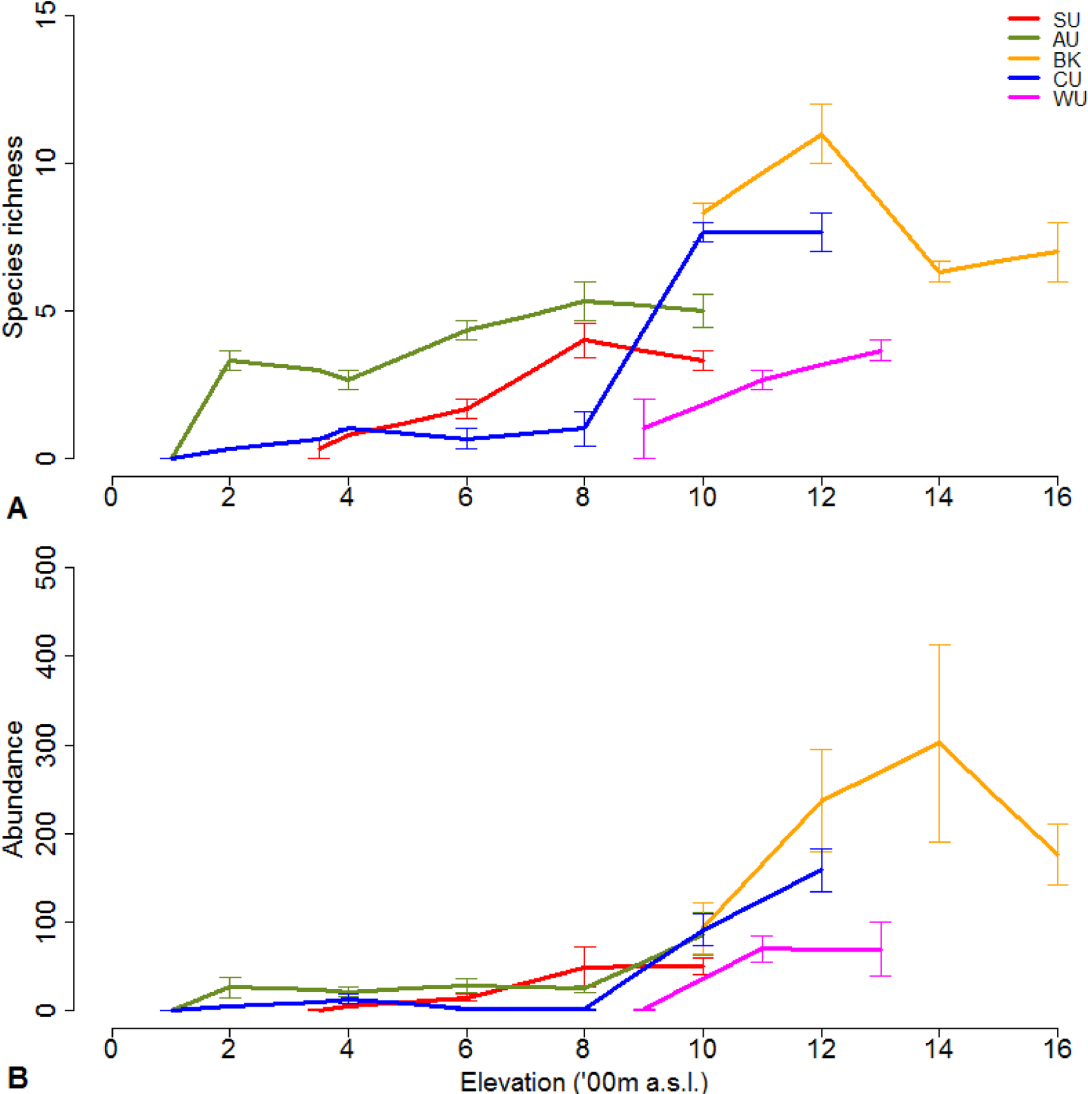
**Changes in flightless ground beetle (a) observed species richness (±SE, n = 3) and (b) abundance (±SE, n = 3) with elevation across elevational gradients.** SU = Spec Uplands; AU = Atherton Uplands; BK = Bellenden Ker Uplands; CU = Carbine Uplands & WU = Windsor Uplands.

Species richness and abundance of flightless ground beetles increased with elevation in the Wet Tropics (Fig 3). Lower elevation zones often entirely lacked flightless ground beetles. Species richness peaked at 1000 and 1200 m a.s.l. in the Carbine Uplands and Bellenden Ker Uplands. Flightless ground beetles were most abundant at Bellenden Ker Uplands, where elevations only at or above 1000 m a.s.l. were sampled. For all other subregions, beetles were consistently low in abundance below 1000 m a.s.l. Controlling for the influence of subregional differences, generalised linear mixed models showed that elevation was significantly and positively related to species richness (estimated coefficient = 0.75, standard error = 0.20, *t* value = 3.72, *P* = 0.0002, n = 69) and, albeit marginally significant, abundance (estimated coefficient = 1.16, standard error = 0.66, *t* value = 1.83, *P* = 0.067, n = 69).

**Fig 4:**
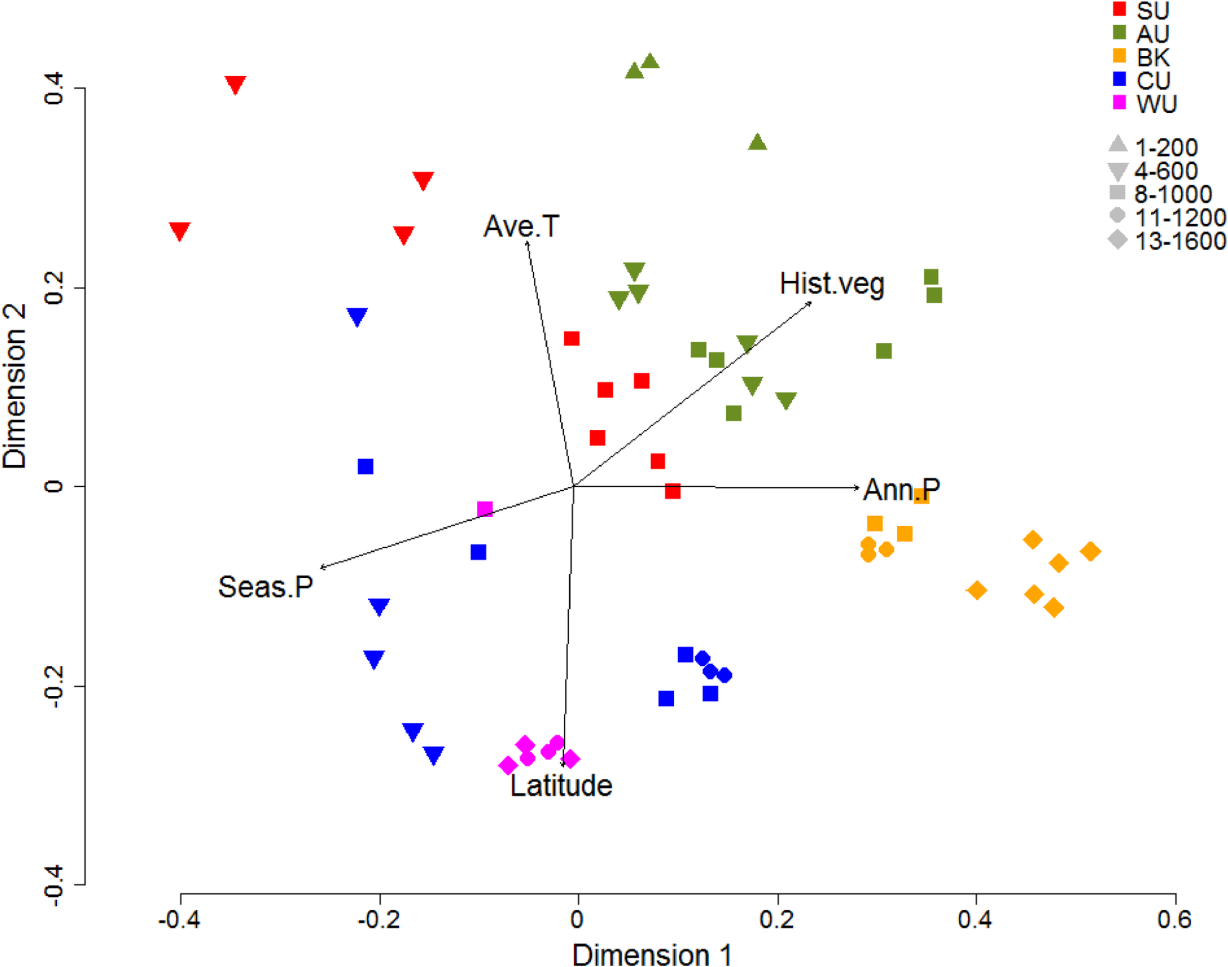
Community composition of flightless ground beetles in the Wet Tropics as described by NMDS ordination. Ordination based on Bray Curtis similarities derived from square-root transformed abundance data from pitfall traps (stress = 0.22). Overlaid vectors are those of the five predictor variables indicated by the information theoretic approach as most likely (*P* value <0.001) to explain variation in the community composition of flightless ground beetles. Seas.P = precipitation seasonality; Ann.P = annual precipitation; Hist.veg = historical vegetation stability; Latitude = plot latitude; Ave.T = annual mean temperature. SU = Spec Uplands; AU = Atherton Uplands; BK = Bellenden Ker Uplands; CU = Carbine Uplands & WU = Windsor Uplands. All of the samples collected at 100 m a.s.l. at AU and CU were excluded as no beetles were collected.

Assemblage composition of flightless beetles was clearly different among the subregions. Subregional assemblages showing the closest affinities were the southern Spec Uplands and central Atherton Uplands, and the northern Carbine Uplands and Windsor Uplands (Fig 4). Within each subregion, however, assemblage composition was elevationally stratified, with samples collected from lower elevations clearly different from those from higher elevations. PERMANOVA statistically confirmed that both subregion (pseudo-*F* = 8.10, *P* < 0.001) and elevation (pseudo-*F* = 9.89, *P* < 0.001) were significantly related to assemblage composition. The five predictor variables, indicated by information theoretic approach as most likely (*P* value <0.001) to explain variation in the community composition of flightless beetles (see below section), were overlaid on the ordination. This analysis indicated that increased latitude and precipitation seasonality are associated with more northern assemblages (Carbine Uplands and Windsor Uplands) at the bottom of the ordination pane whereas increases in average temperature and historical habitat stability are associated with the central and southern assemblages (Atherton and Spec Uplands) at the top of the ordination pane. Increases in annual precipitation appear to be associated with the Bellenden Ker assemblages in the ordination.

### Associations with environmental factors

The information theoretic approach showed that differences in species richness were likely to be explained by the historical stability of vegetation, disturbance from treefalls, annual mean temperature, precipitation seasonality, and marginally, temperature seasonality (Table 2). The highest effect sizes (> 3.0) were achieved by historical stability of vegetation and treefall disturbance. Unlike the climatic variables, these two factors were not highly correlated with elevation (correlation coefficients of 0.03 and -0.34 for historical stability of vegetation and treefall disturbance respectively, see S1 Table).

**Table 2:** Summary results of the information theoretic approach for relationships between predictor variables and *observed species richness* of flightless ground beetles, showing (a) summed Akaike weights from observed data, (b) mean summed Akaike weights from randomised data, (c) standard deviation (SD) of summed Akaike weights, standardised effect size ((a-b)/c), and *P* values calculated from 999 null models generated by permutation. Hist.veg = historical vegetation stability; Treefall = disturbance from treefalls; Ave.T = annual mean temperature; Seas.P = precipitation seasonality; Seas.T = temperature seasonality; Litter = fine-litter standing crop; AWC = available water capacity; TWI = topographic wetness index; Ann.P = annual precipitation; Latitude = plot latitude; Aspect = plot aspect; Habitat = habitat heterogeneity of forest floor. Predictor variables displayed in bold were significant.

Environmental variable	(a) Summed Akaike weight (observed)	(b) Mean summed Akaike weight (null models)	(c) SD of summed Akaike weight (null models)	Standardised effect size	*P* value
**Hist.veg**	0.85	0.36	0.12	4.16	**0.008**
**Treefall**	0.88	0.35	0.15	3.53	**0.010**
**Ave.T**	0.72	0.35	0.13	2.82	**0.031**
**Seas.P**	0.64	0.36	0.12	2.32	**0.046**
Seas.T	0.68	0.36	0.15	2.21	0.056
Litter	0.60	0.36	0.16	1.49	0.097
AWC	0.53	0.35	0.14	1.26	0.099
TWI	0.30	0.36	0.16	-0.38	0.468
Ann.P	0.30	0.36	0.15	-0.38	0.491
Latitude	0.26	0.35	0.14	-0.63	0.695
Aspect	0.25	0.35	0.16	-0.67	0.785
Habitat	0.23	0.36	0.16	-0.80	0.998

**Fig 5:**
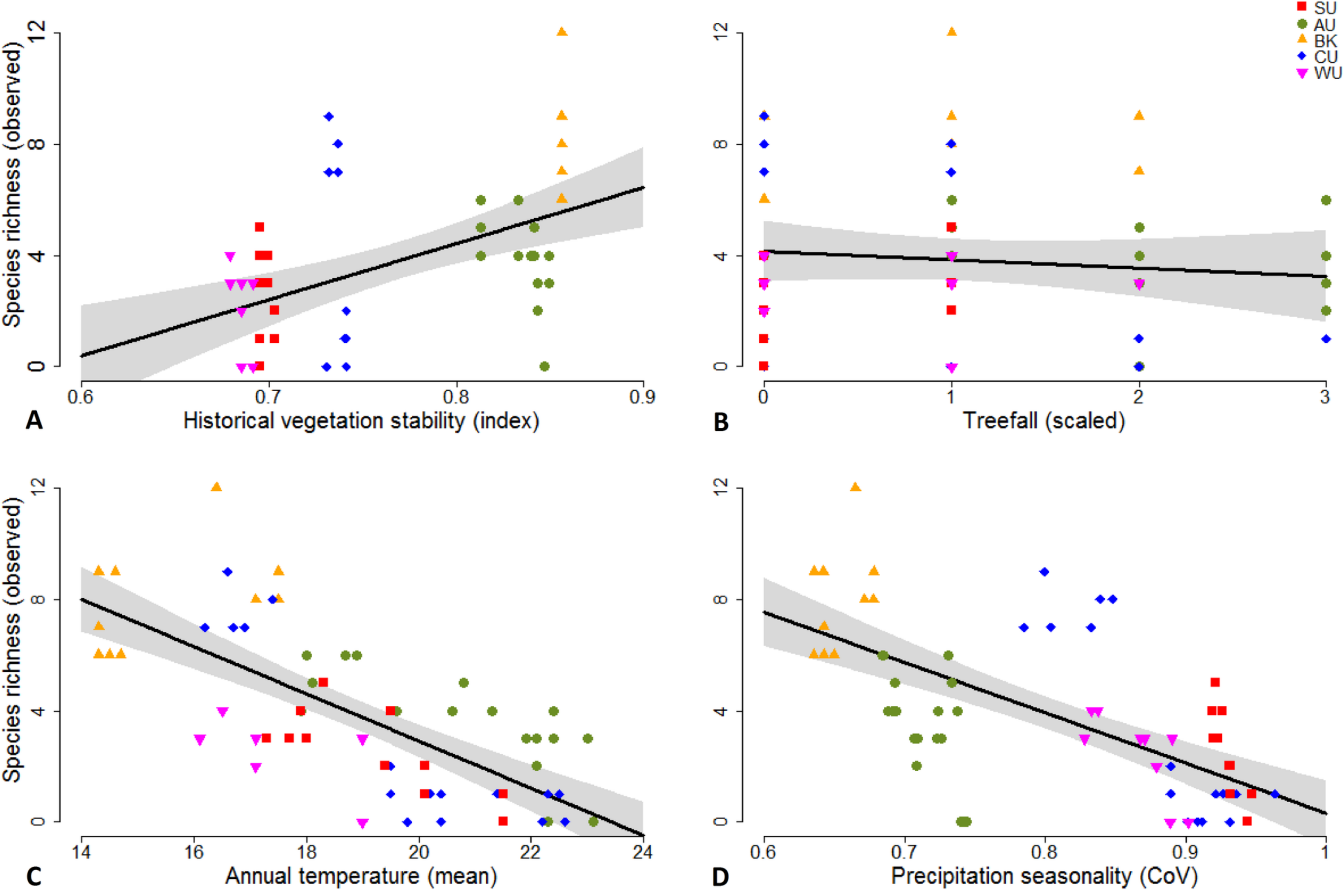
**Correlations between observed species richness and (A) historical vegetation stability, (B) disturbance from treefalls, (C) annual mean temperature and (D) precipitation seasonality, indicated by the information theoretic approach (see Table 2).** SU = Spec Uplands; AU = Atherton Uplands; BK = Bellenden Ker Uplands; CU = Carbine Uplands & WU = Windsor Uplands.

Linear regression (Fig 5) indicated that species richness was positively correlated with the historical stability of vegetation (Adj. *R*^*2*^ = 0.19, *P* < 0.001), and was negatively correlated with annual mean temperature (Adj. *R*^*2*^ = 0.49, *P* < 0.001) and precipitation seasonality (Adj. *R*^*2*^ = 0.40, *P* < 0.001). Species richness did not display a linear relationship with disturbance from treefalls (Adj. *R*^*2*^ = -0.005, *P* = 0.420). The impact of treefall was, however, highly significant when using the information theory (Table 2) which tested models using combinations of different variables.

Differences in assemblage composition were likely to be explained by the environmental variables primarily associated with climatic conditions (Table 3). The highest effect sizes (>14.0) were achieved by current (precipitation seasonality and annual precipitation) and historical (historical stability of vegetation) climatic conditions (Table 3). These plausible variables were not highly correlated with elevation (correlation coefficients of -0.34, 0.45 and 0.03 for precipitation seasonality, annual precipitation and historical stability of vegetation respectively, see S1 Table). However, precipitation seasonality and historical stability of vegetation were highly correlated at correlation coefficient = 0.90.

**Table 3:** Summary results of the information theoretic approach for relationships between predictor variables and *community composition* of flightless beetles, showing (a) summed Akaike weights from observed data, (b) mean summed Akaike weights from randomised data, (c) standard deviation (SD) of summed Akaike weights, standardised effect size ((a-b)/c), and *P* values calculated from 999 null models generated by permutation. Seas.P = precipitation seasonality; Ann.P = annual precipitation; Hist.veg = historical vegetation stability; Latitude = plot latitude; Ave.T = annual mean temperature; Litter = fine-litter standing crop; AWC = available water capacity; Seas.T = temperature seasonality; Treefall = disturbance from treefalls; TWI = topographic wetness index; Aspect = plot aspect; Habitat = habitat heterogeneity of forest floor. Predictor variables displayed in bold were significant.

Environmental variable	(a) Summed Akaike weight (observed)	(b) Mean summed Akaike weight (null models)	(c) SD of summed Akaike weight (null models)	Standardised effect size	*P* value
**Seas.P**	0.67	0.42	0.02	16.19	**<0.001**
**Ann.P**	0.74	0.42	0.02	14.72	**<0.001**
**Hist.veg**	0.64	0.42	0.02	14.70	**<0.001**
**Latitude**	0.63	0.42	0.02	9.89	**<0.001**
**Ave.T**	0.55	0.42	0.02	8.04	**<0.001**
**Litter**	0.55	0.42	0.03	5.18	**0.001**
**AWC**	0.48	0.42	0.02	3.19	**0.009**
**Seas.T**	0.48	0.42	0.02	3.06	**0.009**
**Treefall**	0.48	0.42	0.02	2.77	**0.013**
**TWI**	0.49	0.42	0.03	2.49	**0.024**
**Aspect**	0.47	0.42	0.03	2.17	**0.034**
Habitat	0.37	0.42	0.03	-1.51	0.989

## Discussion

### Sampling effort

The Wet Tropics region contains a highly diverse upland flightless invertebrate community and our study captured the majority of this community’s flightless ground beetle species. Pitfall traps were highly effective at sampling this fauna as indicated by the rarefaction curves which attained a sample coverage of over 99% for all subregions. The majority of known flightless ground beetle species in the region (70% of species from museum records) were sampled using this technique. Flightless ground beetles that were not collected in this study were either very small, and therefore subject to trap bias (*Illaphanus* NQ1, *Sitaphe rotunda*, *Anomotarus* NQ1 and *Feronista* spp.), rare or known to prefer ecotonal habitats (*Notonomus saepistriatus*, *Pamborus elegans*) and therefore unlikely to be sampled with this trap methodology in these locations.

### Elevational patterns across subregions

Species richness of flightless ground beetles generally increased with increasing elevation. This differs from the hump-shaped patterns in richness most commonly displayed by other fauna [10], including the majority of known insect and vertebrate groups throughout the Wet Topics [37, 72]. The species richness and abundance patterns found here reinforce both previous findings by taxonomists [73, 74] and results from species distribution models [38] indicating that these taxa are predominantly distributed throughout upland refugia in the Wet Tropics.

Flightless ground beetle assemblages in the Wet Tropics were highly distinct between subregions. Furthermore, within subregions, assemblages were stratified by elevation. While invertebrates commonly display high levels of assemblage turnover across elevational gradients [75], the fact that this pattern is displayed by a group generally restricted to high elevations attests to their extremely limited distributions. Comparing subregional assemblages, Bellenden Ker Uplands differed substantially from all other locations and the most similar assemblages were those of the Carbine Uplands and Windsor Uplands, and Atherton Uplands and Spec Uplands. Historically, the Bellenden Ker Uplands, Atherton Uplands and Carbine Uplands are thought to have contained among the most stable upland rainforest habitats in the Wet Tropics [76], therefore the distinct assemblages of these subregions are consistent with the time-stability hypothesis [40] where the carabid fauna of these highly stable regions may have undergone extensive isolated speciation.

### Associations with environmental factors

Changes in both the species richness and composition of flightless ground beetles in the Wet Tropics were found to be associated with the historical stability of vegetation. These findings however, come with caveats as precipitation seasonality, another highly plausible predictor variable, was inter-correlated with historical stability of vegetation (correlation coefficient = 0.9, S1 Table). Furthermore, as we do not know the historical dynamics of flightless beetles in this region, we were only able to link current distribution of the beetles with projections of historical stability of vegetation. Although we were unable to quantify the relative importance of current and historical climatic stabilities, our findings are in agreement with previous hypotheses that areas characterised by high historical vegetation stability throughout the Wet Tropics support higher diversities of rainforest-restricted fauna [58], especially taxa with low dispersal abilities [28, 33]. These findings are also consistent with results from a study concerning a Brazilian biodiversity hotspot where a greater diversity of lizards and birds was associated with the historical stability of the region [77] and even a marine ecosystem, where paleodistributions of coral reef fish suggested that historical habitat availability is just as important as current habitat when predicting species richness patterns of these taxa [78].

Flightless ground beetles in tropical regions are thought to have originally colonised lowland regions before dispersing into upland habitats and subsequently losing their ability to fly [34]. Once isolated in stable upland habitats in the Wet Tropics, flightless ground beetles would have been ideal candidates for *in situ* speciation. Previously, *in situ* speciation has been suggested to have driven high levels of endemism among flightless insects within the Wet Tropics [29]. Indeed, a high level of endemism was reported in this study with the proportion of subregional endemics (33%) being more than twice the 15% recorded for co-located vertebrates [25], although, of the vertebrates, microhylid frogs express very high endemicity [79].

Ground beetle diversity is known to be highly sensitive to forest disturbances such as fragmentation, tree thinning and even light gaps [80–83] and this study also suggests that ground beetle community patterns are indirectly affected by disturbances from treefall events. Although linear regression did not display a clear relationship between species richness and disturbance from treefall, the information theoretic approach identified disturbance from treefall to be an important plausible predictor of species richness when assessed within all possible models generated using the 12 predictor variables. Ground beetles species occupying fragmented habitats are often able to be separated into open habitats specialists, forest specialists and habitat generalists [82, 84–86]. As the level of fragmentation in a habitat increases, the proportion of open habitat specialists is known to increase, while habitat generalists persist and forest specialists decrease [80]. It is important to note that prior to this study, a major disturbance event (Cyclone Larry in 2006) dramatically altered vegetation structure and soil properties throughout the Atherton Uplands and it is possible that this event may also have affected our observed diversity patterns by altering the occurrence and/or detection of forest specialists in that area. While the regeneration of these disturbed forests was noted to occur quickly after Cyclone Larry [57, 87] the legacy of the disturbance was still found to negatively impact species richness of flightless ground beetles in this study.

Fine litter standing crop also plausibly explained (albeit not as strongly as current and past climatic predictors) variation in flightless ground beetle composition throughout the Wet Tropics. Research from temperate regions has previously suggested that variations in the litter standing crop affect ground beetle assemblage structure in boreal forests [88]. Throughout the Wet Tropics, litter processes have been linked to disturbance events whereby reduced canopy cover creates higher densities of immature plants resulting in lowered litterfall rates [87]. Indeed, this negative relationship between disturbance from treefall events and litter standing crop is likely to be reflected in the current study and is therefore proposed to influence compositional patterns of flightless ground beetles in the Wet Tropics.

A suite of current climatic variables were also suggested as plausible predictors of variation in the species richness and community composition of flightless ground beetles in the Wet Tropics. This suggests that flightless ground beetle assemblages are also likely to be linked to current climatic conditions throughout this landscape. Greater richness was plausibly explained by upland habitats characterised by cool, stable current climatic conditions. Previous research has linked current climatic variables, such as stable moisture conditions, to greater richness and abundance of ground beetles at high elevations [20, 89]. Additionally, the current findings support other research indicating that ground beetle composition is highly sensitive to current climatic conditions, such as temperature and precipitation [90–92] and that brachypterous species are sensitive to soil water balance [33]. These findings also support other regional invertebrate studies which indicated that, within the Wet Tropics, assemblages of schizophoran flies change in relation to variations in both annual mean temperature and precipitation seasonality [57, 93]. As climate change continues to alter climatic conditions throughout the Wet Tropics, species restricted to high elevations are likely to be impacted by increased temperature and reduced precipitation (due to reduced cloud stripping) [94, 95]. It is likely, therefore, that both the richness and community composition of highly sensitive invertebrates in this region such as flightless ground beetles will change.

## Conclusion

Flightless ground beetles are more species-rich in mountain-top habitats characterised by cool stable climates. These upland communities are highly spatially structured with distinct assemblages between subregional blocks and elevational stratification within each block. The diversity patterns observed were most plausibly explained by the influence of historical vegetation stability, recent disturbance events (treefalls) and current climatic factors such as temperature and precipitation. These findings provide empirical support for previous suggestions that variation in this group’s community composition is strongly linked to stable, upland habitats [38]. Consequently, these findings further support suggestions that community patterns are likely to be influenced by the time-stability hypothesis whereby habitat stability increases the rate of *in situ* evolution of species [40].

The species of this flightless upland group are highly unlikely to disperse across the Wet Tropics’ latitudinal gradient in response to climate change and dispersal is expected only upwards in elevation. Therefore, for the vast majority of species in this group, which are already distributed within the mountain tops, further migration is limited. The current results indicate that this group prefers cool, stable environments and, although the Wet Tropics region has been identified to contain refugia from climate change impacts [96], climatic niches suitable for flightless ground beetles may not exist in this area in the future [38]. Local conservation measures aimed at maintaining microrefugia may be required to buffer these species from intolerable climatic conditions [97]. Additionally, more drastic measures such as assisted dispersal [98, 99], whereby species could be relocated to suitable cooler refugia, may be necessary to prevent the diversity of these flightless ground beetle species from declining and potentially impacting ecosystem functioning. This study clearly highlights the sensitivity of such insects to environmental factors and warrants further research to effectively manage impacts from climate change and maintain the unique biodiversity of this World Heritage Area.

## Supporting Information

S1 Fig Individual-based rarefaction and extrapolation curves for each subregion. (DOCX)

S2 Fig Coverage-based rarefaction and extrapolation curves for each subregion. (DOCX)

S1 Table Pearson correlation coefficients and P values of the 17 predictor variables. (DOCX)

S2 Table Summary of flightless ground beetle species. (DOCX)
