# Supplementary material for: Elevational Distribution of Flightless Ground Beetles in the Tropical Rainforests of North-Eastern Australia
Source: PLoS One. 2016 May 18;11(5):e0155826. doi: 10.1371/journal.pone.0155826 (PMC4871570; doi:10.1371/journal.pone.0155826)
Supplement: S1 Fig — (DOCX) [file pone.0155826.s001.docx]

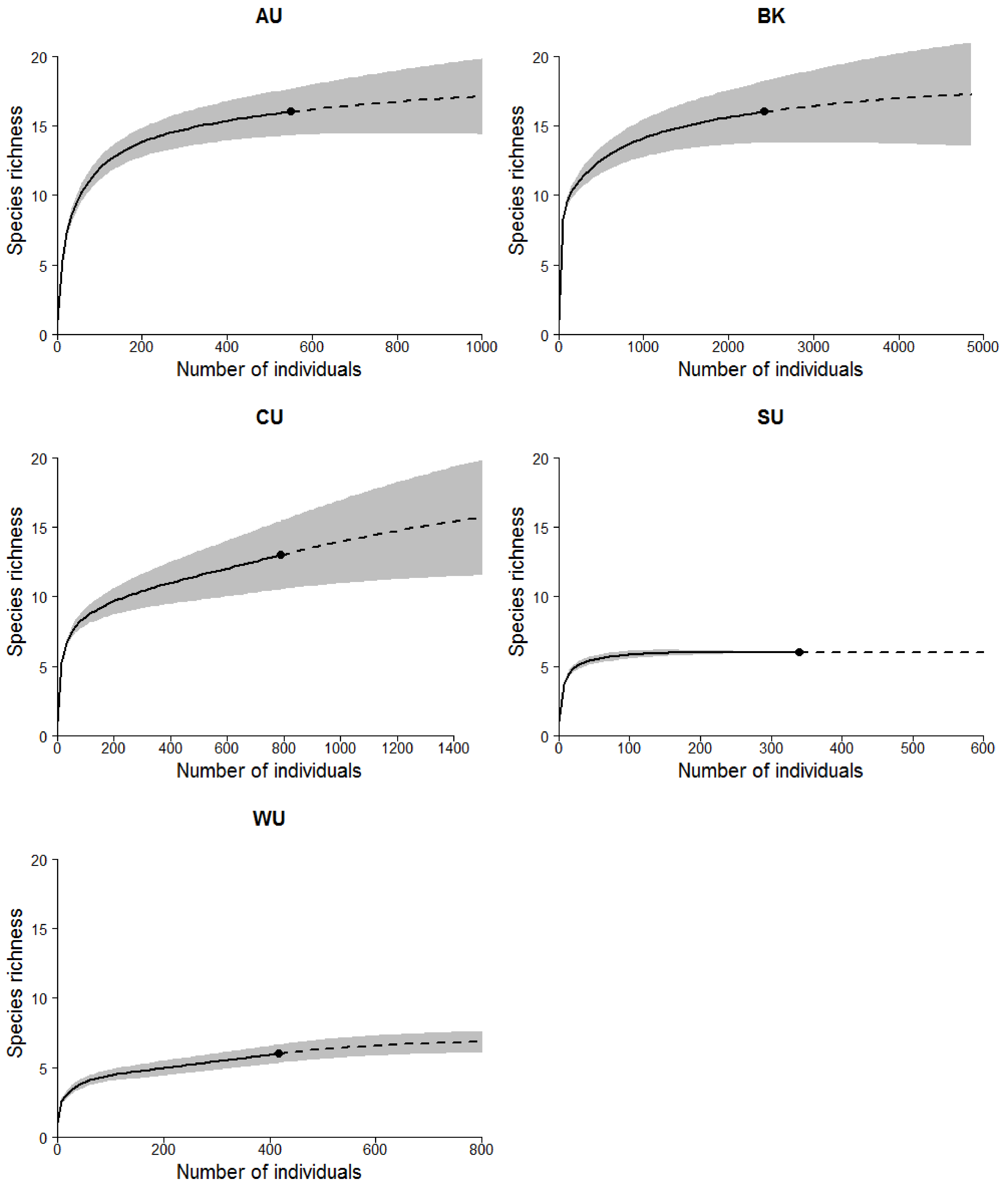


##### **Supplementary material Appendix 1, Fig. A1.** Individual-based rarefaction and extrapolation curves for each subregion – (a) Atherton Uplands, (b) Bellenden Ker Uplands, (c) Carbine Uplands, (d) Spec Uplands, (e) Windsor Uplands. Black circles represent the sampling extent and dashed lines are extrapolations by a factor of 2 with 95% confidence intervals (grey area).
