# Supplementary material for: Elevational Distribution of Flightless Ground Beetles in the Tropical Rainforests of North-Eastern Australia
Source: PLoS One. 2016 May 18;11(5):e0155826. doi: 10.1371/journal.pone.0155826 (PMC4871570; doi:10.1371/journal.pone.0155826)
Supplement: S2 Fig — (DOCX) [file pone.0155826.s002.docx]

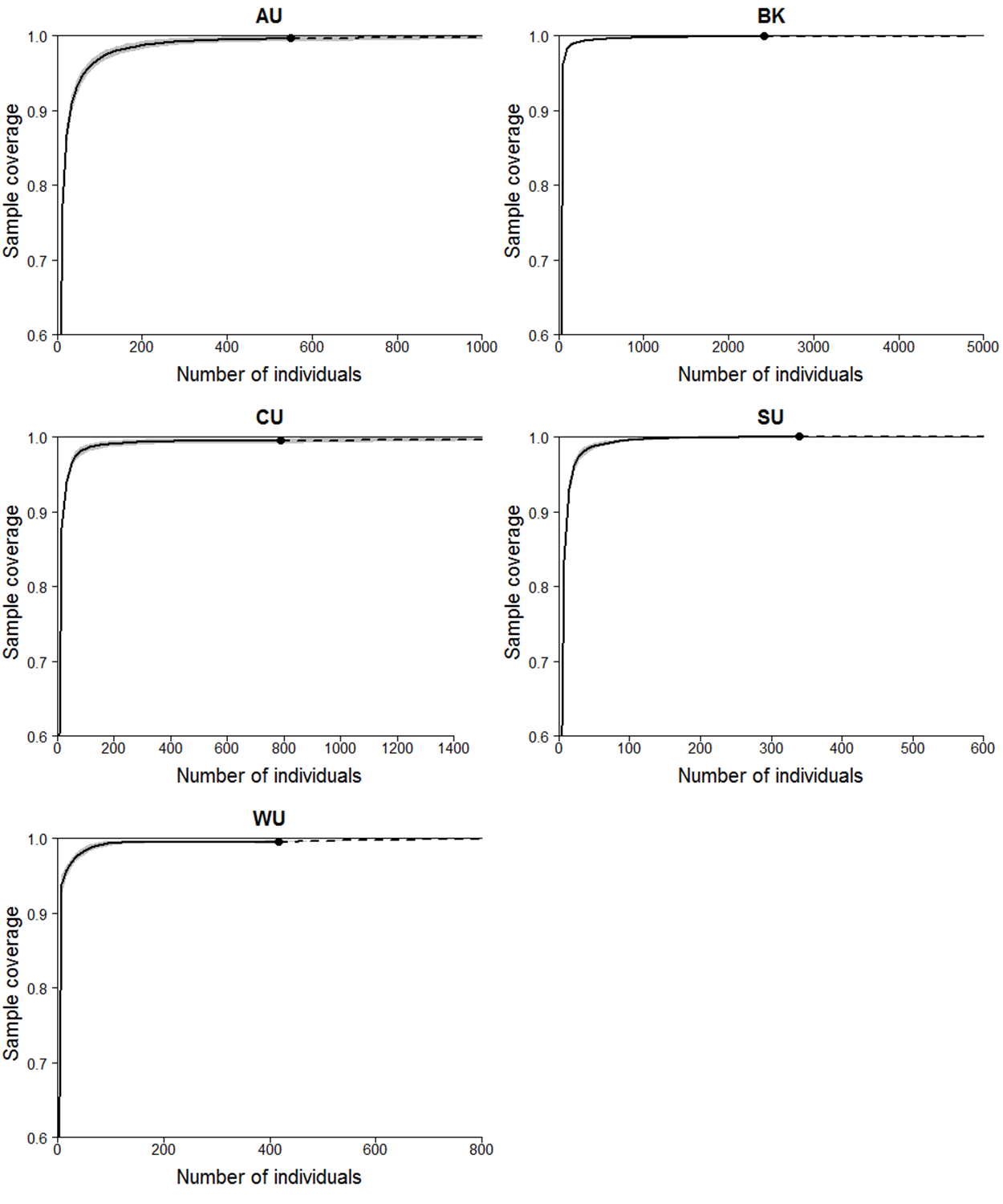


##### **Supplementary material Appendix 1, Fig. A2.** Coverage-based rarefaction and extrapolation curves for each subregion – (a) Atherton Uplands, (b) Bellenden Ker Uplands, (c) Carbine Uplands, (d) Spec Uplands, (e) Windsor Uplands. Black circles represent the sampling extent and dashed lines are extrapolations by a factor of 2 with 95% confidence intervals (grey area). Horizontal lines are drawn where coverage reaches 100% (1.0). Sample coverage of greater than 99% was attained at all of the subregions.
