# Supplementary material for: Elevational Distribution of Flightless Ground Beetles in the Tropical Rainforests of North-Eastern Australia
Source: PLoS One. 2016 May 18;11(5):e0155826. doi: 10.1371/journal.pone.0155826 (PMC4871570; doi:10.1371/journal.pone.0155826)
Supplement: S1 Table — (DOCX) [file pone.0155826.s003.docx]

| Environmental variable | Lat. | Elev.† | Ave.T | Seas.T | Max.T† | Min.T† | Ann.P | Seas.P | P.WetQ† | P.DryQ† | Habitat | Treefall | Litter | Aspect | Hist. veg | | AWC | | TWI |
| --- | --- | --- | --- | --- | --- | --- | --- | --- | --- | --- | --- | --- | --- | --- | --- | --- | --- | --- | --- |
| Lat. |  | **0.050** | 0.166 | **>0.001** | 0.142 | 0.649 | 0.610 | 0.646 | 0.467 | 0.802 | 0.746 | 0.658 | 0.672 | 0.176 | 0.731 | **0.016** | | 0.763 | |
| Elev.† | 0.24 |  | **>0.001** | **0.001** | **>0.001** | **>0.001** | **>0.001** | **0.004** | **>0.001** | **>0.001** | **0.012** | **0.004** | **>0.001** | 0.053 | 0.820 | 0.142 | | **>0.001** | |
| Ave.T | -0.17 | **-0.97** |  | **>0.001** | **>0.001** | **>0.001** | **>0.001** | **0.004** | **>0.001** | **>0.001** | **0.018** | **0.002** | **>0.001** | 0.053 | 0.728 | 0.257 | | **0.001** | |
| Seas.T | -0.50 | -0.40 | 0.45 |  | **>0.001** | 0.192 | 0.115 | 0.598 | 0.055 | 0.222 | 0.940 | **0.007** | 0.146 | 0.488 | 0.198 | 0.427 | | **0.018** | |
| Max.T† | -0.18 | **-0.92** | **0.97** | 0.61 |  | **>0.001** | **>0.001** | 0.067 | **>0.001** | **>0.001** | **0.044** | **>0.001** | **>0.001** | 0.131 | 0.600 | 0.496 | | **>0.001** | |
| Min.T† | -0.06 | **-0.94** | **0.94** | 0.16 | 0.85 |  | **>0.001** | **0.002** | **>0.001** | **>0.001** | **0.007** | **0.011** | **>0.001** | 0.227 | 0.735 | **0.024** | | **0.012** | |
| Ann.P | 0.06 | 0.45 | -0.49 | -0.19 | -0.41 | -0.44 |  | **>0.001** | **>0.001** | **>0.001** | 0.068 | 0.486 | 0.261 | 0.077 | **>0.001** | 0.226 | | **0.009** | |
| Seas.P | -0.06 | -0.34 | 0.34 | -0.06 | 0.22 | 0.37 | -0.80 |  | **>0.001** | **>0.001** | 0.078 | **0.012** | 0.434 | **0.009** | **>0.001** | 0.854 | | 0.231 | |
| P.WetQ† | 0.09 | 0.45 | -0.49 | -0.23 | -0.43 | -0.44 | **1.00** | -0.76 |  | **>0.001** | 0.078 | 0.638 | 0.290 | 0.115 | **>0.001** | 0.223 | | **0.008** | |
| P.DryQ† | 0.03 | 0.47 | -0.50 | -0.15 | -0.41 | -0.47 | **0.99** | -0.83 | **0.98** |  | **0.047** | 0.415 | 0.212 | **0.042** | **>0.001** | 0.224 | | **0.006** | |
| Habitat | 0.04 | -0.30 | 0.28 | -0.01 | 0.24 | 0.32 | -0.22 | 0.21 | -0.21 | -0.24 |  | 0.423 | 0.566 | 0.831 | 0.228 | 0.617 | | 0.266 | |
| Treefall | 0.05 | -0.34 | 0.37 | 0.32 | 0.43 | 0.30 | 0.09 | -0.30 | 0.06 | 0.10 | 0.10 |  | **>0.001** | 0.371 | **>0.001** | 0.333 | | 0.065 | |
| Litter | 0.05 | 0.47 | -0.47 | -0.18 | -0.43 | -0.47 | 0.14 | -0.10 | 0.13 | 0.15 | -0.07 | -0.41 |  | 0.264 | 0.447 | 0.265 | | 0.880 | |
| Aspect | 0.16 | 0.23 | -0.23 | -0.08 | -0.18 | -0.15 | 0.21 | -0.31 | 0.19 | 0.25 | -0.03 | 0.11 | 0.14 |  | **0.004** | **0.043** | | **0.034** | |
| Hist.veg | -0.04 | 0.03 | -0.04 | 0.16 | 0.06 | -0.04 | 0.73 | -0.90 | 0.68 | 0.76 | -0.15 | 0.43 | -0.09 | 0.34 |  | 0.064 | | 0.212 | |
| AWC | 0.29 | 0.18 | -0.14 | 0.10 | -0.08 | -0.27 | -0.15 | -0.02 | -0.15 | -0.15 | -0.06 | 0.12 | 0.14 | -0.24 | -0.22 |  | | **0.001** | |
| TWI | -0.04 | -0.42 | 0.41 | 0.29 | 0.42 | 0.30 | -0.31 | 0.15 | -0.32 | -0.33 | 0.14 | 0.22 | -0.02 | -0.26 | -0.15 | 0.38 | |  | |

##### **Supplementary material Appendix 1, Table A1.** Pearson correlation coefficients (bottom left triangle) and P values (top right triangle) of the 17 predictor variables. Five predictor variables (denoted by †) were removed from subsequent analysis due to high collinearity with other predictors (r>0.90). Correlation coefficients greater than 0.90 and significant P values (<0.05) highlighted in bold. Lat. = site latitude; Elev. = site elevation; Ave.T = annual mean temperature; Seas.T = temperature seasonality; Max.T = maximum temperature of warmest period; Min.T = minimum temperature of coldest period; Ann.P = annual precipitation; Seas.P = precipitation seasonality; P.WetQ = precipitation of the wettest quarter; P.DryQ = precipitation of the driest quarter; Hab. = habitat heterogeneity of forest floor; T. fall = disturbance from treefalls; Litter = fine-litter standing crop; Aspect = site aspect; Hist.veg = historical vegetation stability; AWC = available water capacity; TWI = total wetness index.
