# Supplementary material for: Elevational Distribution of Flightless Ground Beetles in the Tropical Rainforests of North-Eastern Australia
Source: PLoS One. 2016 May 18;11(5):e0155826. doi: 10.1371/journal.pone.0155826 (PMC4871570; doi:10.1371/journal.pone.0155826)
Supplement: S2 Table — (DOCX) [file pone.0155826.s004.docx]

##### **Supplementary material Appendix 1, Table A2.** Table of flightless ground beetle species sampled indicating subregional endemics Y=Yes, N=No, total abundance and elevational range (’00 m a.s.l.) of species sampled within each subregion. Spec Uplands (SU), Atherton Uplands (AU), Bellenden Ker Uplands (BK), Carbine Uplands (CU), Windsor Uplands (WU).

| Species | Subregion Endemic | SU | Range | AU | Range | BK | Range | CU | Range | WU | Range |
| --- | --- | --- | --- | --- | --- | --- | --- | --- | --- | --- | --- |
| *Castelnaudia obscuripennis* | N |  |  |  |  |  |  | 45 | 4-12 | 165 | 9-13 |
| *Castelnaudia setosiceps* | N |  |  | 7 | 4-10 | 63 | 10-12 |  |  |  |  |
| *Castelnaudia* sp.1 | Y | 16 | 3.5-6 |  |  |  |  |  |  |  |  |
| *Castelnaudia spec* | N | 46 | 6-10 |  |  |  |  |  |  |  |  |
| *Coptocarpus* NQ1 | N |  |  |  |  |  |  | 1 | 12 |  |  |
| *Coptocarpus philipi* | N |  |  |  |  | 1 | 12 |  |  |  |  |
| *Craspedophorus* sp.1 | Y |  |  | 6 | 2-4 |  |  |  |  |  |  |
| *Feronista* sp.1 | Y |  |  |  |  | 4 | 10-16 |  |  |  |  |
| *Feronista* sp.2 | Y |  |  |  |  |  |  | 6 | 10-12 |  |  |
| *Feronista* sp.3 | Y |  |  |  |  | 3 | 12 |  |  |  |  |
| *Laccopterum* sp.1 | N |  |  | 19 | 8 |  |  |  |  |  |  |
| *Laccopterum* sp.2 | N |  |  | 3 | 4 |  |  |  |  |  |  |
| *Lecanomerus limbatus* | N |  |  | 2 | 8 |  |  |  |  |  |  |
| *Lecanomerus niger* | N |  |  | 1 | 8 | 7 | 10-14 |  |  |  |  |
| *Lecanomerus* sp.1 | N |  |  |  |  |  |  | 1 | 12 |  |  |
| *Leiradira alternans* | N |  |  | 8 | 6-10 |  |  |  |  |  |  |
| *Leiradira alticola* | Y |  |  |  |  | 217 | 10-16 |  |  |  |  |
| *Leiradira* NQ2 | N |  |  |  |  |  |  | 6 | 10-12 |  |  |
| *Leiradira opacistriatus* | N |  |  | 5 | 6 |  |  |  |  |  |  |
| *Leiradira soror* | N |  |  |  |  | 8 | 12 |  |  |  |  |
| *Mecyclothorax storeyi* | N |  |  |  |  | 2 | 14 |  |  |  |  |
| *Mystropomus regularis* | N | 73 | 8-10 | 20 | 6-10 | 507 | 10-16 | 140 | 8-12 |  |  |
| *Notonomus dimorphicus* | Y |  |  |  |  |  |  | 46 | 10-12 |  |  |
| *Notonomus doddi* | N | 5 | 8-10 |  |  |  |  | 1 | 8 | 13 | 11-13 |
| *Notonomus flos* | Y |  |  |  |  |  |  | 77 | 10-12 |  |  |
| *Notonomus masculinus* | N |  |  | 71 | 6-10 |  |  |  |  |  |  |
| *Notonomus montellus* | Y |  |  |  |  | 28 | 10-16 |  |  |  |  |
| *Notonomus montorum* | N |  |  |  |  | 811 | 10-16 |  |  |  |  |
| *Notonomus* NQ1 | Y |  |  |  |  |  |  |  |  | 17 | 9-13 |
| *Notonomus spurgeoni* | N |  |  |  |  |  |  | 26 | 10-12 |  |  |
| *Oodes* sp.1 | N |  |  | 14 | 2-4 |  |  | 1 | 6 |  |  |
| *Pamborus euopacus* | N |  |  |  |  |  |  | 418 | 10-12 | 221 | 11-13 |
| *Pamborus punctatus* | N |  |  | 1 | 10 | 64 | 10-16 |  |  |  |  |
| *Pamborus tropicus* | N | 150 | 8-10 | 258 | 4-8 |  |  |  |  | 1 | 9 |
| *Pheropsophus verticalis* | N |  |  | 59 | 2 |  |  |  |  |  |  |
| *Prosopogmus* sp. 2 | Y |  |  |  |  |  |  |  |  | 1 | 9 |
| *Setalis rubripes* | N |  |  |  |  | 1 | 12 |  |  |  |  |
| *Trichosternus fax* | Y |  |  |  |  | 297 | 14-16 |  |  |  |  |
| *Trichosternus frater* | N |  |  |  |  |  |  | 24 | 10-12 |  |  |
| *Trichosternus montorum* | Y |  |  |  |  | 337 | 10-16 |  |  |  |  |
| *Trichosternus mutatus* | Y |  |  | 56 | 10 |  |  |  |  |  |  |
| *Trichosternus nudipes* | N | 50 | 6-10 |  |  |  |  |  |  |  |  |
| *Trichosternus soror* | N |  |  | 21 | 6-8 | 78 | 10-12 |  |  |  |  |
| Total abundance |  | 340 |  | 551 |  | 2,428 |  | 792 |  | 418 |  |
